# Methylated Poly(ethylene)imine Modified Capacitive Micromachined Ultrasonic Transducer for Measurements of CO_2_ and SO_2_ in Their Mixtures

**DOI:** 10.3390/s19143236

**Published:** 2019-07-23

**Authors:** Dovydas Barauskas, Donatas Pelenis, Gailius Vanagas, Darius Viržonis, Jonas Baltrušaitis

**Affiliations:** 1 Panevėžys faculty of Technologies and Business, Kaunas University of Technology, Nemuno st. 33-218, Panevėžys 37164, Lithuania; 2 Department of Chemical and Biomolecular Engineering, Lehigh University, B336 Iacocca Hall, 111 Research Drive, Bethlehem, PA 18015, USA

**Keywords:** CMUT, methylated polyethyleneimine, CO_2_, SO_2_, gravimetric detection, gas sensing

## Abstract

A gravimetric gas detection device based on surface functionalized Capacitive Micromachined Ultrasound Transducers (CMUTs) was designed, fabricated and tested for detection of carbon dioxide (CO_2_) and sulfur dioxide (SO_2_) mixtures in nitrogen. The created measurement setup of continuous data collection, integrated with an in-situ Fourier Transform Infrared (FT-IR) spectroscopy, allows for better understanding of the mechanisms and molecular interactions with the sensing layer (methylated poly(ethylene)imine) and its need of surface functionalization for multiple gas detection. During experimentation with CO_2_ gases, weak molecular interactions were observed in spectroscopy data. Linear sensor response to frequency shift was observed with CO_2_ concentrations ranging from 0.16 vol % to 1 vol %. Moreover, the Raman and FT-IR spectroscopy data showed much stronger SO_2_ and the polymer interactions, molecules were bound by stronger forces and irreversibly changed the polymer film properties. However, the sensor change in resonance frequency in the tested region of 1 vol % to 5 vol % SO_2_ showed a linear response. This effect changed not only the device resonance frequency but also affected the magnitude of electroacoustic impedance which was used for differentiating the gas mixture of CO_2_, SO_2_, in dry N_2_.

## 1. Introduction

The constant increase in gas pollution generating industrial activities, such as the burning of fossil fuels or animal husbandry, has increased emissions of sulfur, carbon and nitrogen oxides into the atmosphere [1,2,3]. High concentrations of sulfur dioxide and carbon dioxide can be found near petroleum refineries, commodity chemical industries, power plants and ore processing plants. Increasing concentrations of these gases in the environment are associated with health hazards that can cause respiratory illnesses, smog, acid rain or accumulation and growth of aerosol particles [1,4]. This necessitates an increased focus on monitoring and controlling these emissions by developing gas sensing systems that are size and cost-effective, sensitive and selective towards particular acidic gases.

Routinely, detection of gas molecule mixtures in the air can be performed by dedicated analysis systems based on infrared spectroscopy [5,6,7], gas chromatography [8,9], mass spectrometry or gravimetry [10]. Some of these methods result in bulky and expensive devices that use a lot of power and require routine maintenance/calibration, are incompatible with integrated circuits (ICs) and cannot readily be scaled down. On the other hand, resonant gravimetric techniques can be miniaturized and use little power while still maintaining the selectivity and sensitivity required [11]. Some of these have already been used for different applications and have the potential for integration into mobile applications. Some examples of such sensors based on resonant gravimetry include film bulk acoustic resonators (FBARs) [12,13,14], quartz crystal microbalance (QCM) [15,16,17,18], surface acoustic wave (SAW) sensors [19,20,21,22], micro and nanocantilevers [23,24,25,26,27] and Capacitive Micromachined Ultrasound Transducers (CMUTs) [28,29,30,31,32,33]. CMUTs can be fabricated in different sizes and shapes with optimized features fulfilling needed mass sensitivity of the resonator, the limit of detection and signal to noise ratio (SNR). CMUT’s are composed of an array of resonating structures, usually referred to as the CMUT cells, that can operate in parallel. When several of the membranes are damaged or not working properly, other membranes can still provide useful data regarding the obtained signal. Furthermore, the active sensing area is much higher and miniaturization potential and integration to other electronics is higher when compared to another type of gravimetric sensor.

CMUTs have been used for dimethyl methylphosphonate (DMMP) [30,31,34], water, ethanol, acetone, ethyl acetate, methane [33] and carbon dioxide in air [35], biomolecule [32,36] and carbon dioxide in nitrogen [37,38] detection. Multiple gases can be detected by functionalizing the CMUT surface with materials, usually, polymers, that interact with specific analytes [39]. This kind of surface modification can be performed using spin coating [40,41], spray coating [42], dip coating [43], ink jet printing [44] and layer by layer (LBL) deposition [45]. It has been shown that an increase in selectivity can be achieved by coating different CMUT elements with different sensing layers [33,37,46]. Some researchers showed a possibility of making wireless, low power, CMUT-based gas sensors with designs including multiple channels for better selectivity [39,47,48,49]. However, sensor functionalization, while resulting in selective molecular interactions with the analyte, can result in very strong binding and irreversible changes to the molecular structure.

In this work, a gas sensor suitable for acidic gas (CO_2_ and SO_2_) mixture detection is presented. The sensor, consisting of the capacitive micromachined ultrasound transducer, is described and detailed in this work together with the experimental testing setup. The sensor was spin-coated with a thin layer of a polymer that interacts with the gases of interest. Most of the similar CMUT gas sensor systems mainly focus their measurements on the resonant oscillations as a way to read the sensor. In this work, a functional material methylated poly(ethylene)imine (mPEI) is utilized for developing highly selective sensors towards SO_2_ and CO_2_. The selected functional material interacts with gas molecules differently resulting not only in the change in the resonance frequency of the CMUT but it also affects the magnitude of the real part of the electroacoustic impedance. The measurement of both parameters leads to selective detection of target gases in the gas mixture. Supporting in-situ infrared spectroscopy measurements of the gas spectral information were collected. The experimental data of the sensing element response towards CO_2_ and SO_2_ with particular emphasis on sensing of their mixtures are shown. Fundamental interactions of these gases with the functionalization mPEI layer are described using infrared spectroscopy methods including in-situ Fourier Transform Infrared spectroscopy and ex-situ Raman spectroscopy.

## 2. Materials and Methods

### 2.1. CMUT Device Analysis, Design, Fabrication and Assembly

To analyze and find the optimal operating point and working conditions an analytical CMUT model using an equivalent circuit method [50,51,52,53,54] was utilized. In this model, all of the CMUT cells are regarded as mechanical resonators. The resonance frequency of such cells, consisting of top and bottom electrodes and a vacuum gap in between, can be expressed as

(1) $$f=\frac{1}{2\pi }\sqrt{\frac{k_{s}-k_{e}}{m_{m}+m_{a}}}$$

here, *k_s_*—elastic coefficient of the membrane, *k_e_*—elastic coefficient of the membrane due to electrostatic force, *m_m_*—mass of the membrane, *m_a_*—any additional mass on the surface of the membrane.

The elastic coefficient, *k_s_*, of the membrane depends on its shape and can be calculated using Hooke’s law, having a square shaped membrane with a side length of 2*a_m_*

(2) $$k_{s}=\frac{188D}{a_{m}^{2}}\;\mathrm{where}\;D=\frac{Ed_{m}^{3}}{12\left(1-\nu^{2}\right)}$$

here, *E*—Young’s modulus of the membrane material, *v—*Poisson’s ratio of the membrane material, *d_m_*—membrane thickness.

Under non-ideal conditions, the oscillations of the membrane are attenuated due to the structural and environmental factors. The total attenuation coefficient can be calculated with Equation (3) by the algebraic sum of the structural attenuation, *b_vs_,* and the environmental attenuation, *b_va._*

(3) $$b_{v}=b_{vs}+b_{va}$$

The main attenuation originating from environmental factors, such as temperature, pressure or humidity, can be calculated, assuming that the sensing layer is a liquid substance, from Equation (4)

(4) $$b_{va}=Z_{s}\frac{A_{m}^{2}}{A_{v}}$$

here, *A_m_*—an area of the membrane, *A_v_*—full area of the transducer including the area which does not vibrate, Z_s_—acoustic impedance of liquid substance expressed as

(5) $$Z_{s}=\sqrt{\rho_{s}\left[\frac{1}{\xi_{s}}+j\omega \left(2\eta_{2}+\eta_{1}\right)\right]}\approx \rho_{s}c_{s}$$

here, *ρ_s—_*density of the membrane material, *ξ_s_*—compressibility of adiabatic gas, *c_s_*—the speed of sound in the gas, *η_1_* and *η_2_*—first and second viscosity coefficients of gas.

When the attenuation coefficient is known, it is possible to calculate the quality factor, Q, from an equivalent circuit model

(6) $$Q=\frac{f_{0}}{\Delta f}=2\pi f_{0}\frac{m_{m}}{b_{vs}}$$

here, *Δf—*width of resonance frequency peak at the level of −3 dB. 

For CMUT, the quality factor usually does not reach above 100, using the most popular fabrication methods, such as wafer bonding and sacrificial release [37,55,56]. However, when thin plates and very thin vacuum gaps are formed, it is possible to obtain quality factors over 100 [57,58] and other researchers have also shown an impressive quality factor of 400 [34].

**Figure 1:**
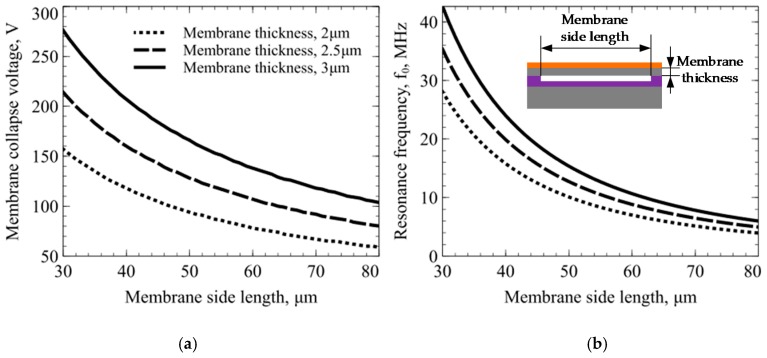
Capacitive micromachined ultrasonic transducer (CMUT) analytical model results: (**a**) membrane collapse voltage as a function of membrane side length and thickness; (**b**) membrane resonance frequency as a function of membrane side length and thickness. A small inset shows the structure of the single CMUT cell and the parameters simulated in the analytical model.

CMUT design parameters are given in Table 1, which was established in previous work [59]. The thickness of the membrane can be determined by the SOI wafers device layer thickness. However, a custom-order of SOI wafers with required thickness and parameters is expensive; the two options already available in the market, with 2 to 3 µm device layer, were used. Using the analytical model, two main functions were obtained regarding the membrane size, collapse voltage, resonance frequency and plate thickness. Figure 1 shows the analytical model results of collapse voltage and resonance frequency as a function of the membrane size and thickness. Using analytical data, an optimized design point was selected based on factors such as collapse voltage and maximum sensing area. The sensing area was limited to 4 mm^2^ and the pull-in voltage to be below 200 V. Based on these two limitations the lateral dimension of the membrane was selected to be 42 μm. In the given limited sensing area it was possible to fit 40 capacitive cells every 50 µm with 8 µm space in between creating a full array of 1600 cells in total. Based on the analytical results, the selected side length of the capacitive cells corresponds to a resonance frequency at ~16 MHz with the membrane thickness of 2 μm.

**Figure 2:**
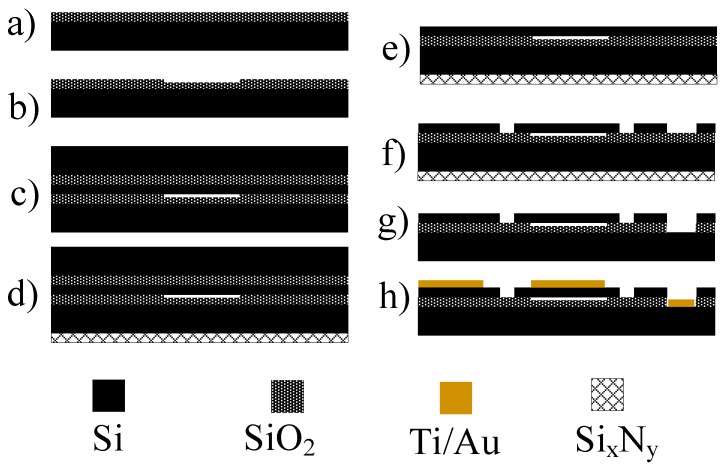
CMUT wafer bonding fabrication steps: (**a**) Thermal oxidation; (**b**) oxide wet etching; (**c**) wafer bonding; (**d**) backside protection with plasma enhanced chemical vapor deposition (PECVD) silicon nitride; (**e**) handle wafer removal using CMP and wet etch; (**f**) Oxford cryogenic etching process for separating devices by etching device layer; (**g**) opening of contact pads with reactive ion etch (RIE), (**h**) top electrode formation and contact pads metallization using lift-off procedure.

**Figure 3:**
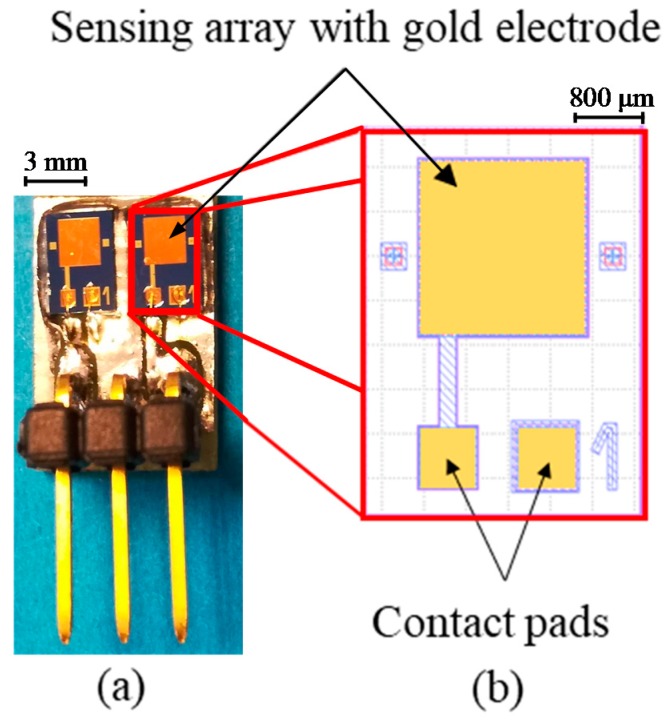
(**a**) Two CMUT chips assembled on a custom printed circuit board with connection pins and contact pads bonded to the printed circuit board (PCB) with gold wires, (**b**) design of the CMUT chip.

Gravimetric CMUT sensor design was based on the obtained data from the analytical model. Fabrication parameters used for the membrane size, thickness, number of cells, resonance frequency and collapse voltage are shown in Table 2 as obtained in previous work [59]. CMUT prototype chips were fabricated in microfabrication cleanroom ISO Class 5 facilities using the wafer bonding technique [60,61] with standard microfabrication processes. The wafer bonding process allowed creating devices with controllable properties of the design parameters such as the depth of the vacuum gap, moving plate thickness, membrane size and shape. Figure 2 shows the processing steps used for CMUT prototype chip microfabrication. First, 4 inch Si wafers with 300 nm of thermally grown SiO_2_ was patterned using UV photolithography with positive photoresist. Patterned wafers were submerged into a buffered oxide etch (BOE) solution. The etching rates of BOE are known and are used widely [62,63,64,65]. This etch step created cavities for the vacuum gap of the designed CMUT device. Cavity depth was controlled by timing of the buffered oxide etch step. Etch depth was optimized by measuring the depth of the cavities using ellipsometer Woollam M2000. The wafers with etched cavities were bonded to silicon on insulator (SOI) wafers with a layer thickness of 2 µm (refer to Figure 2c). Cavities were sealed under vacuum during the fusion bonding process. Prior to the bonding process, all wafers underwent an RCA cleaning to remove any particles that could create voids during bonding delaminating the substrate wafer from the SOI wafer [66]. In this case, the device (top) layer of the SOI wafer determines the membrane thickness. Later, the back side of the wafers was covered with a 200 nm layer of PECVD Si_x_N_y_ for protection during wet etching step (Figure 2d) since the selectivity of PECVD SiN and the KOH solution is high. The handle wafer (bottom layer of SOI wafer) was then chemically–mechanically polished (CMP) using a 400BC grit slurry solution and Logitech PM-5 Lapping/CMP equipment. The remainder were etched using 40 wt. % potassium hydroxide (KOH) solution at 80 °C. Another UV photolithography step was used to separate the devices by etching Si through the device layer (Figure 2e).For this purpose, a cryogenic Oxford Si etch process was used [67,68], creating almost straight and perpendicular sidewalls separating different chips. Last, the UV photolithography step was used to form the top electrode and to metalize the contact pads. This included the use of negative photoresist, since after the development the pattern has negative sidewalls, which help in the lift-off procedure. For forming the top electrode, Lesker™ Evaporator was used, depositing a thin 25 nm Ti layer, which acted as the adhesion between the silicon substrate and gold, and 175 nm Au layer. A lift-off procedure was done after evaporation in an ultrasonic bath of resist remover for 2 hours. Finally, the wafers were diced to separate the individual CMUT chips and these chips were assembled by gluing the prototype chips on a custom printed circuit board and connecting the contact pads and the traces of the PCB with 50 μm gold wires using the ultrasonic welding technique. For protection, the main connections were covered using transparent epoxy resin. The design of the CMUT chip and fully assembled devices on a printed circuit board with custom connection pins are shown in Figure 3a,b respectively.

### 2.2. CMUT Surface Functionalization

For the specific binding interaction with the acidic gas molecules, CMUT chips were functionalized with a polymer—methylated poly(ethylene-imine) (mPEI) synthesized as described previously [69,70]. In numerous previous works, poly(ethylene-imine) (PEI) was tested and shown to be a good absorber of CO_2_ [37,71,72,73,74]. Methylated PEI (PEI solution with the primary and secondary amines converted to tertiary) was also shown to be a good absorber of SO_2_, while also capable of capturing CO_2_ under humid conditions [70,75]. Prior to functionalization, the devices were cleaned with ethanol and dried with dry N_2_ stream. mPEI was diluted with a solution of methyl alcohol (MeOH) and tetrahydrofuran (THF) to decrease the viscosity of the polymer film and to be able to spin coat a moderately thin layer on top of the CMUT sensing zone. The coating was performed using a VTC-100 vacuum spin coater by pre-spinning the diluted polymer solution at 200 rpm for 5 seconds to obtain a continuous polymer layer, followed by ramping up to 800 rpm and spinning for 60 seconds to thin out the formed layer.

### 2.3. CMUT Resonance Frequency Characterization

**Figure 4:**
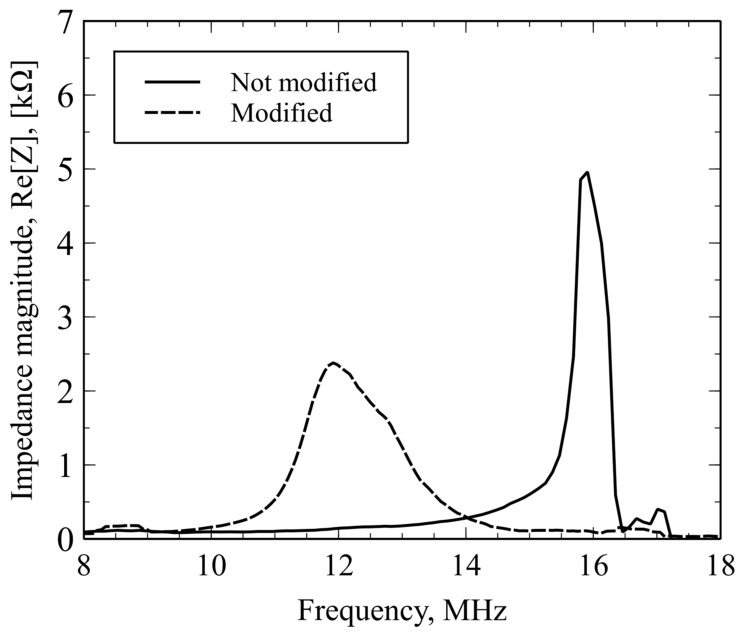
Impedance magnitude spectra of the CMUT device before and after spin-coated methylated poly(ethylene-imine) (mPEI) layer as a function of frequency.

Gravimetric detection principles rely on the measurement and tracking of the resonance frequency of the resonating structure. When CMUT array is spin-coated by a thin layer of polymer, its resonance frequency decreases due to the added mass. The resonance frequency shift after surface functionalization with mPEI was determined using a Network analyzer Agilent 4395A equipped with an impedance measurement kit. The resonance frequency was determined from frequency spectra of the real part of the impedance that was acquired by the network analyzer. To reduce the noise and amplify output for the resonance frequency detection, a Colpitts type oscillator circuit that uses CMUT as an electromechanical resonator [37,76,77] was used. As seen in Figure 3a, two sensing chips were connected. One of the sensors was uniformly covered with the mPEI polymer and another was used as a reference without the polymer. This was done to subtract any additional noise coming from the environment, such as variations in temperature, humidity, pressure or flow rate. When using two CMUT elements in parallel, better SNR values can be achieved than using a single chip. The fabricated and fully assembled CMUT devices were characterized before and after spin coating with the thin mPEI polymer film (refer to Figure 4) and the resonance frequency for the fabricated devices decreased by ~4 MHz due to the added additional mass onto the vibrating membrane. Since the thin spin-coated polymer film was viscous, the magnitude of the device impedance spectrum was also reduced [52,78].

## 3. Experimental Setup

### In-Situ Infrared Spectroscopy Measurement Setup

**Figure 5:**
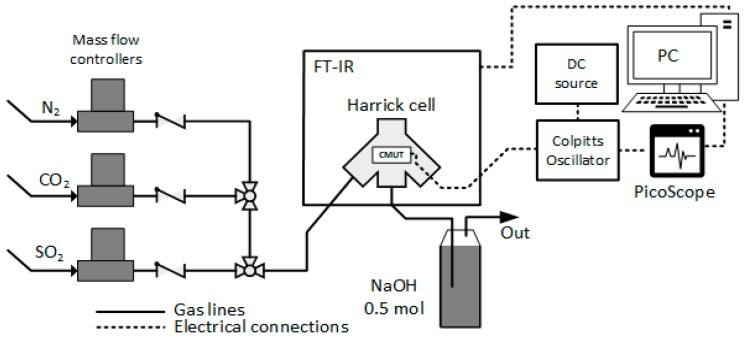
Experimental setup for in-situ simultaneous real-time CMUT resonance frequency and the magnitude of electroacoustic impedance measurement and Fourier transform infrared spectroscopy [59].

Measurement setup for the simultaneous real-time resonance frequency detection and Fourier transform infrared spectroscopy is shown in Figure 5 and is described in more detail in previous work [59]. Briefly, with this setup, the control and set concentrations of CO_2_, SO_2_ and N_2_ gases were achieved using Brooks GF40 series precision mass flow controllers which were calibrated for each gas. Dry N_2_ was used for purging the reaction chamber and for the dilution of CO_2_ and SO_2_ to test lower concentration values. Software from Brooks Instruments was used to set a specified gas flow rate and set the specific concentration. All of the gas mixtures after use were neutralized using a bubbler filled with 0.5 mol NaOH solution.

During the experiments, fully assembled CMUT chips were placed inside the closed reaction chamber (a modified version of Harrick Praying Mantis^TM^ diffuse reflectance accessory) within an FT-IR spectroscope (ThermoFisher iS50) and connected using sealed feedthrough to the Colpitts oscillator circuit [79]. The oscillator circuit was driven by an external power supply unit for DC source while the high bias voltage for the optimal operating point was generated from a custom low noise external power source circuit. The analog signal from the oscillator circuit was fed into a PicoScope^TM^ oscilloscope. The oscilloscope was set to a data streaming mode for real-time signal acquisition and registration of resonance frequency and impedance magnitude. The oscilloscope was connected to the PC using Universal Serial Bus (USB) connection. Data were collected with MATLAB script and PicoLog toolset in order to control the acquisition rate and integrate it into other software. MatLab was used as the analysis software for ease of calculations and data interpretation. The measurements of Fourier transform infrared spectroscopy, CMUT resonant frequency and the magnitude of electroacoustic impedance were monitored and saved simultaneously in real time. 

During the experimentation with various gas flow rates and preset concentration values, the testing chamber was always firstly flushed with dry N_2_ for 30 minutes with a preset flow rate of 100 mL/min before introduction of any specific gas concentration and after the experiment the chamber was flushed again with N_2_ for 60 minutes at the same flow rate. The flushing steps ensured that the CMUT prototype chips would stabilize and reach an equilibrium point. All of the experiments were performed at room temperature with concentrations ranging from 0.16 to 1.6 vol % for CO_2_ and from 1 to 5 vol % for SO_2_.

## 4. Results

### 4.1. Real-Time Cross-Selective Gas Sensing Experiment Results

**Figure 6:**
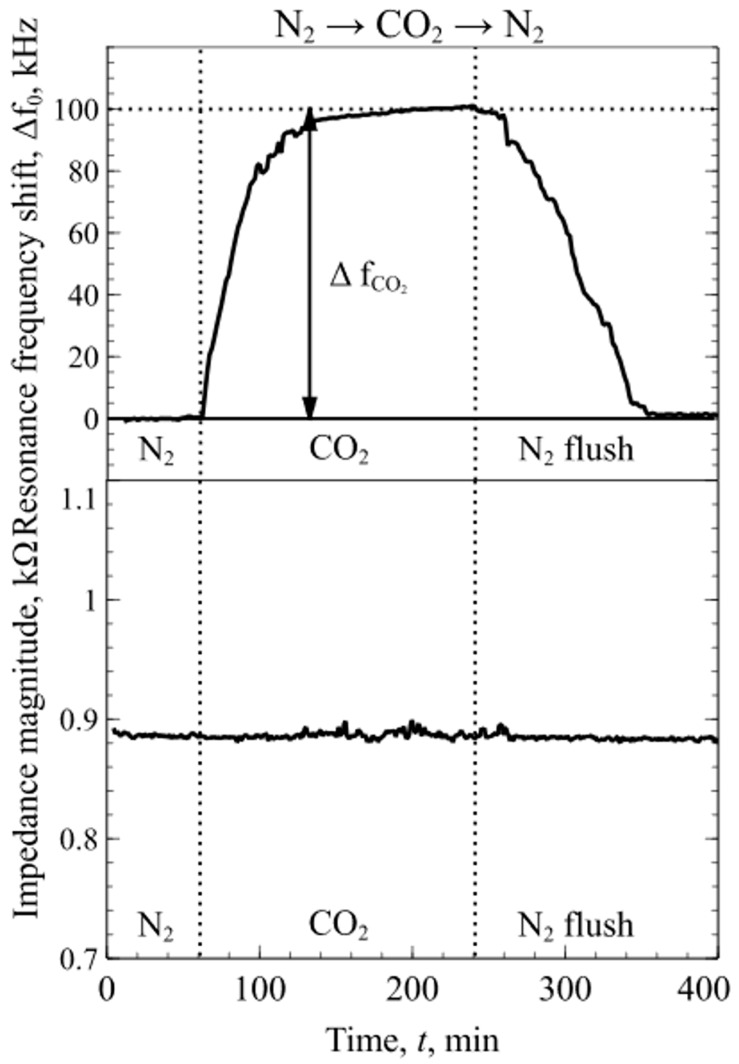
Continuous resonance frequency shift and magnitude of the impedance spectra as a function of time when transitioning between N_2_ and CO_2_.

During the sensing experiment, when transitioning between the dry N_2_ to a 1.6 vol % concentration of CO_2_ and back to N_2_, an apparent resonance frequency shift, Δf_CO2_, was detected while the magnitude of the electroacoustic impedance did not exhibit any detectable changes. Relating to Equation (1), any additional mass on the membrane changes its resonance frequency and, according to Equation (6), its quality factor, which is directly related to the impedance magnitude. Since the resonance frequency shift is small (<0.4% from *f_0_*)*,* the change in magnitude of the impedance spectra is in the noise level range and could not be measured. Flushing the testing chamber with dry N_2_ returned the resonance frequency values to the previously measured levels, as seen in Figure 6. These results suggest that CO_2_ molecules are very weakly bound (physisorbed) and mPEI does not make any strong chemical interactions with CO_2_. 

**Figure 7:**
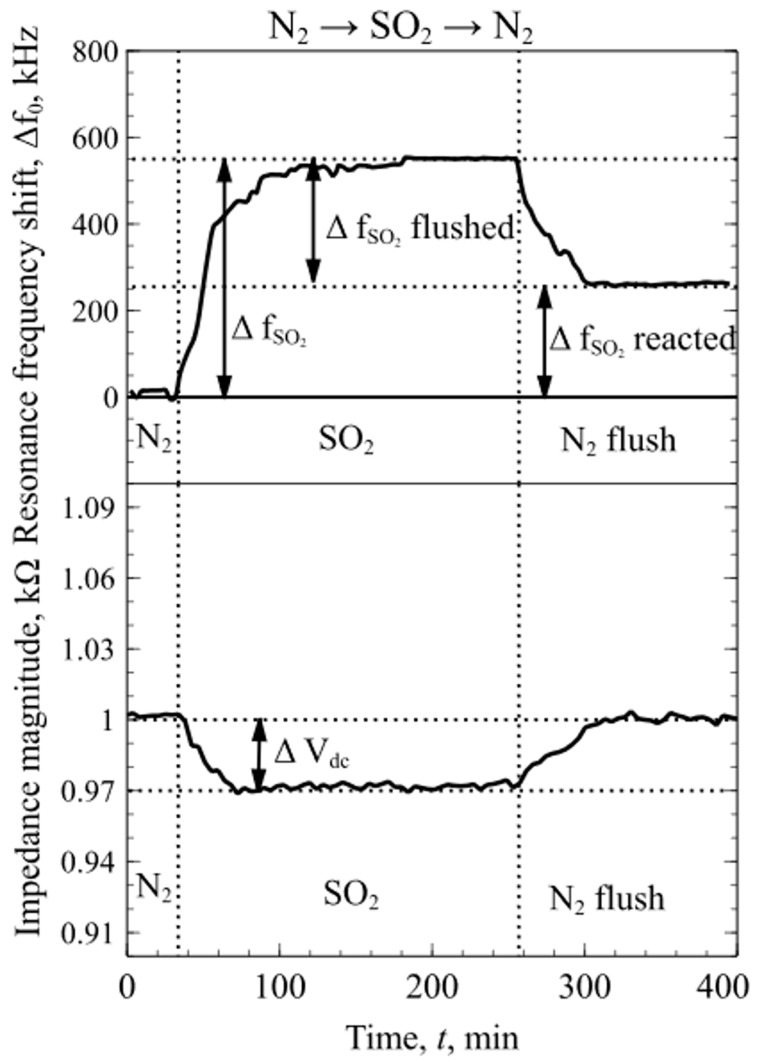
Continuous resonance frequency shift and magnitude of the impedance spectra as a function of time when transitioning between N_2_ and SO_2_.

When performing an N_2_ diluted SO_2_ experiment sensing with a concentration of 5 vol %, shifts in both resonance frequency and magnitude of the impedance spectra were observed. According to Equation (6), the shift in signal magnitude is related to the changes in membrane mass, *m_m_*, and attenuation coefficient, *b_vs_*. Due to the strong interactions between the surface functionalized CMUT/mPEI and SO_2_ molecules, the physical structure of the functional polymer film changes the membrane mass and attenuation coefficient, which induces high resonance frequency shift and the decrease of impedance magnitude as it is shown in Figure 7. After flushing the chamber with dry N_2_, the impedance magnitude returned to previous values while the resonance frequency did not, relating to the mass increase by the reacted mPEI and SO_2_.

**Figure 8:**
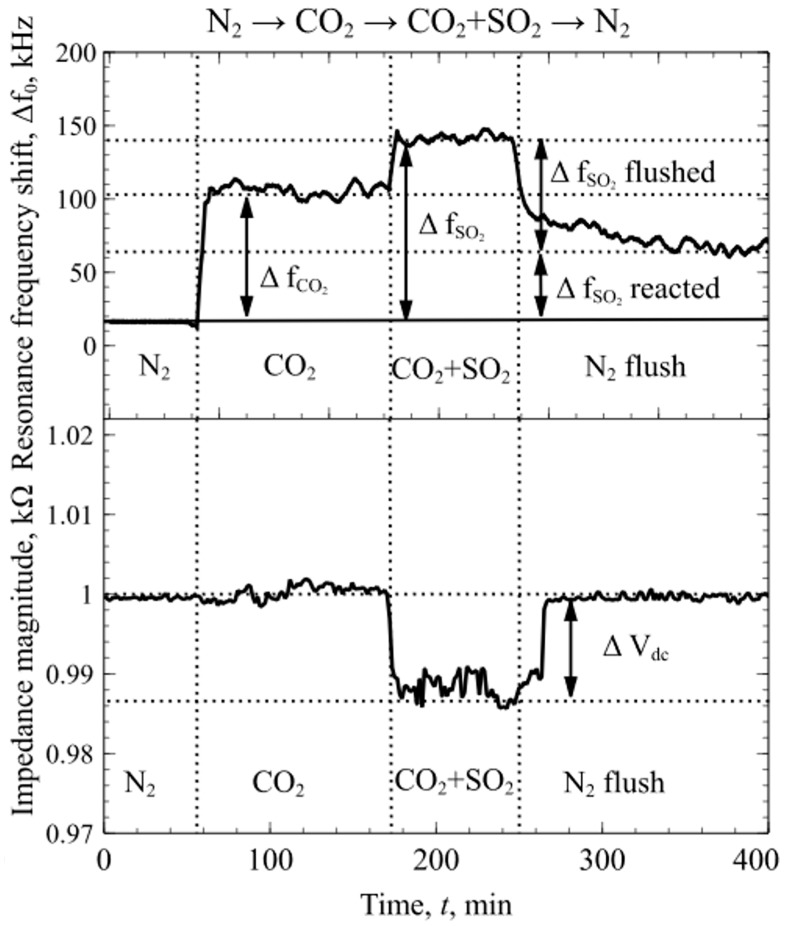
Continuous resonance frequency shift and magnitude of the impedance spectra as a function of time when transitioning between N_2_, CO_2_ and CO_2_ + SO_2_ mixture.

Lastly, experiments with the mixtures of dry N_2_, CO_2_ and SO_2_ were conducted to determine the changes in mPEI functionalized CMUT chip resonance frequency and magnitude of electroacoustic impedance. First, the test chamber was flushed with a constant flow rate of dry N_2_ for the sensors to reach an equilibrium state. The resonance frequency and impedance magnitude were measured in real time and logged continuously during the experiment. When CO_2_ was introduced into the chamber, due to the weak interactions between the mPEI and CO_2_ molecules, the resonance frequency shift, Δf_CO2_, was observed without any apparent detectable change in the magnitude of the impedance spectra. When SO_2_ was introduced into the mixture, due to a strong chemical interaction between the mPEI and SO_2_ molecules, the changes in resonance frequency and the magnitude of the impedance were observed at the point when SO_2_ was introduced and reacted with the mPEI layer, as shown in Figure 8. This phenomenon can be related to the weak CO_2_ and mPEI interactions, which do not change the physical structure of the polymer, but the strong chemical interactions with SO_2_ molecules change the properties of the polymer film. Following Equations (1) and (6), these changes increase the attenuation coefficient and mass which in turn leads to the shift in resonance frequency and decrease in impedance magnitude that can be seen in Figure 8. The difference in the resonance frequency shift shown in Figure 7 and Figure 8 for SO_2_ is due to the fact that SO_2_ were diluted together with CO_2_ in dry N_2_, leading to a much lower concentration than in the previous experiment. Importantly, the results presented in Figure 6, Figure 7 and Figure 8 suggest that, by considering changes in both resonance frequency and impedance, a mixture of two acidic gases, SO_2_ and CO_2_, can be detected and contributions of each gas can be decoupled.

### 4.2. Resonance Frequency Shift Measurement Results after CO_2_ and SO_2_ Interaction with mPEI Functionalized CMUT

**Figure 9:**
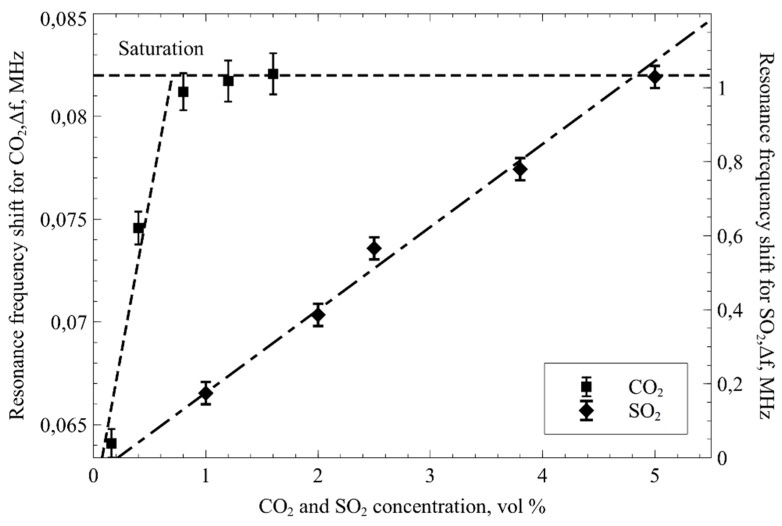
The recorded resonance frequency shift of mPEI covered CMUT as a function of CO_2_ concentration and inert N_2_ at 23 °C (square data points). CMUT, covered with a thin layer of mPEI, resonance frequency dynamics with different SO_2_ concentrations and inert N_2_ at 23 °C (diamond data points).

The average resonance frequency shift of multiple tested devices at various concentrations of CO_2_, ranging from 0.16 vol % to 1.6 vol % are shown in Figure 9. Two different adsorption regimes have been observed. First, a linear regime with R^2^ = 0.928 was observed at lower CO_2_ concentrations. In this region, an apparent limit of detection (LOD) was calculated using LOD = 3.3 σ/slope with an RMSE value of 0.911 as an estimate for σ. The LOD was found to be 0.011 CO_2_ vol %, while the limit of quantification (LOQ) was calculated to be 0.033 CO_2_ vol %. The measured sensitivity was 8 Hz/ppm. Similar CO_2_ adsorption regimes were observed previously [38,59]. Note that the concentrations of the CO_2_ were calculated as a ratio between the CO_2_ and N_2_ flows set by the precision mass flow controllers.

As with CO_2_, the resonance frequency shifts were also observed when SO_2_ were introduced into the testing chamber (Figure 8). The difference from the CO_2_ gas tests is that SO_2_ exhibited linear response with R^2^ = 0.9899 at SO_2_ concentrations ranging from 1 to 5 SO_2_ vol %. For this region, the limit of detection was calculated the same way as for CO_2_ LOD = 3.3 σ/slope with an RMSE value of 0.2127 as an estimate for σ. The LOD was found to be 0.232 SO_2_ vol % while the limit of quantification was found to be 0.704 SO_2_ vol %. The measured sensitivity for SO_2_ was calculated to be 20 Hz/ppm. All of the data points were acquired by doing a pulse of different gas concentrations ranging from 0.16 vol % to 1.6 vol % for CO_2_ and 1 vol % to 5 vol % for SO_2_.

### 4.3. Mass Sensitivity of mPEI Functionalized CMUT

Gravimetric detection principles rely on their ability to detect small amounts of mass and mass sensitivity is one of the most important parameters for a sensing system based on the gravimetric detection principle. Approximating the CMUT as a one-dimensional linear harmonic oscillator, it is possible to calculate the theoretical distributed mass sensitivity assuming that the adsorbed mass by the polymer is quantitively much smaller than the plate mass [28]

(7) $$S_{theoretical}=\frac{\delta f_{0}}{\delta m}=-\frac{1}{2}\frac{f_{0}}{m_{plate}}$$

here, *f_0_* is the resonance frequency of the device and *m_plate_* is the mass of the plate. 

When the resonance frequency is at the designed 16 MHz, the theoretical mass sensitivity for the fabricated devices was calculated as 0.536 Hz/fg.

### 4.4. In-Situ DRIFTS Fourier Transform Infrared Spectroscopy and Ex-Situ Raman Spectroscopy of CMUT Devices Employing a Thin mPEI Film

**Figure 10:**
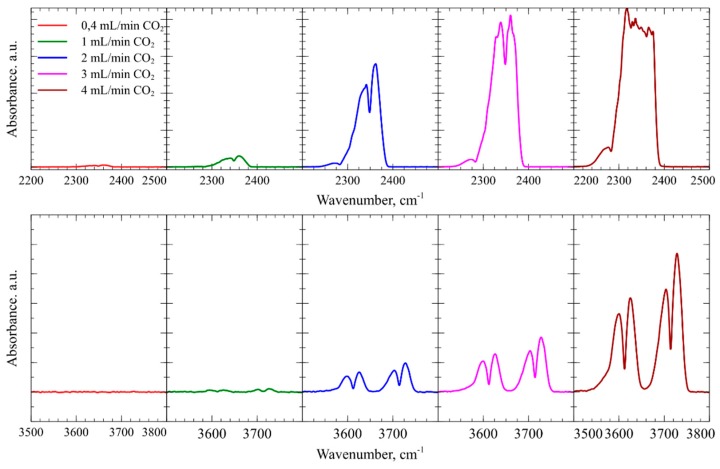
Fourier Transform Infrared spectroscopy data peaks formed at wavenumbers in region 2200 to 2500 cm^−1^ and region 3500 to 3800 cm^−1^ produced by absorbance of CO_2_ phase molecules.

**Figure 11:**
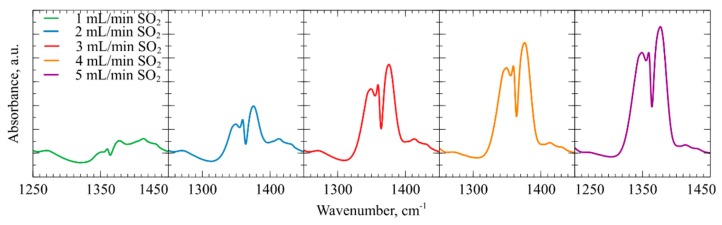
Fourier Transform Infrared spectroscopy data peaks formed at wavenumbers in region 1250 to 1450 cm^−1^ produced by absorbance of SO_2_ phase molecules.

During the experiments with CO_2_ and SO_2_, the analysis system collected FT-IR spectra in real time. Figure 10 shows absorbance peak zones produced by the CO_2_ at various flow rates. In the spectral region from wavenumber 2200 to 2500 cm^−1^ due to the asymmetric stretch of gaseous CO_2_ and the region from wavenumber 3500 to 3800 cm^−1^, the produced two doublet bands correspond to the overtones of CO_2_ vibrational bands. In Figure 11 infrared absorption peaks are produced by symmetric and asymmetric stretching of SO_2_ molecules in the wavenumber region from 1250 to 1450 cm^−1^ [80]. 

**Figure 12:**
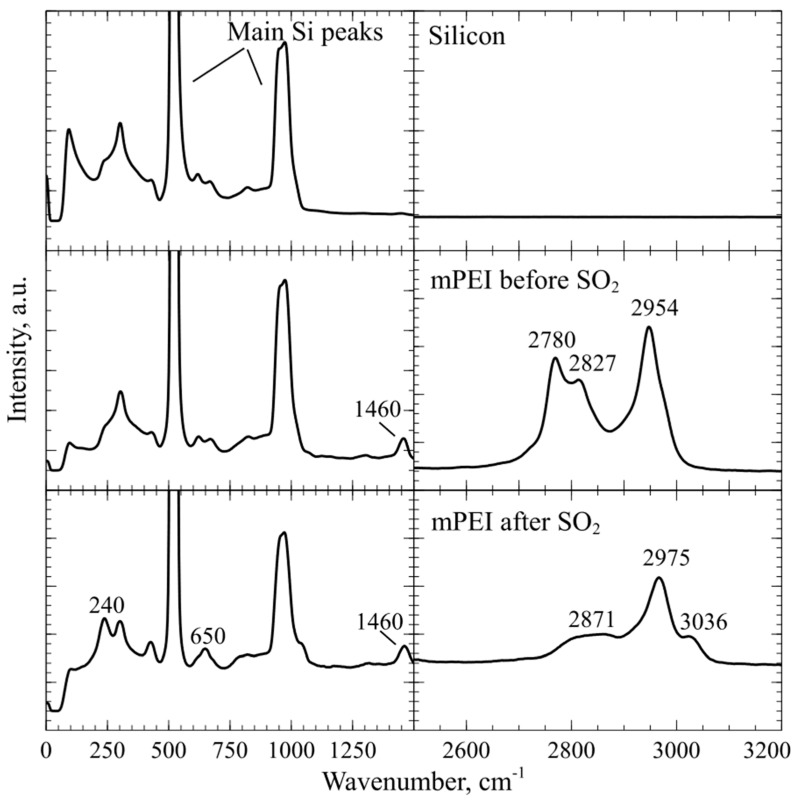
Raman spectra comparison of Si, Si with spin coated thin layer of mPEI and Si with a thin layer of mPEI after SO_2_ gas experiments [59].

The designed in-situ DRIFTS measurement setup can be used to get more information regarding gas interaction with the sensing polymer layer. From the experiments with CO_2_ gas FTIR spectroscopy, it was found that, due to the mPEI not making any strong chemical bonds, CO_2_ molecules are only physisorbed within the mPEI structure. The data shown in Figure 9 suggests that CO_2_ binding mode can be reversible at concentrations below 0.8 vol %. However, FTIR spectroscopy and experiments with SO_2_ gases showed an irreversible adsorption behavior, but a highly linear response through the whole tested concentration range. SO_2_ can irreversibly adsorb onto the mPEI functional layer, changing the properties of the layer. These changes can be clearly seen in Figure 12**,** where a surface functionalized CMUT with mPEI was tested using ex-situ Raman spectroscopy. These spectra were collected before and after the device interacted with the SO_2_ gases. After mPEI film interacted with SO_2_, the intensity of the peaks below the wavenumber 2850 cm^−1^ decreased. A new peak was observed at wavenumber 3036 cm^−1^. When SO_2_ binds to the amine, the symmetric C-H stretching vibrations at a wavenumber below 2850 cm^−1^, which are associated with methyl groups, disappear and C-H stretching vibrations create an intensity peak at wavenumber ~3030 cm^−1^ [70]. The ex-situ Raman spectroscopy data agrees well with the experimental data from other measurements since the SO_2_ binding to the functional layer affects the physical structure of the polymer film the resonance frequency shifts permanently, even after flushing the testing chamber with dry N_2_ and the impedance magnitude measurements show changes at the point when SO_2_ starts to bind to the mPEI layer. Since CO_2_ is known to bind weakly via physical interactions that do not change the magnitude on the impedance over time, but SO_2_ molecules bind more strongly to mPEI through chemical bonds. This bonding affects the performance of the device which in turn can be measured and used as a detection channel.

From the experimental results, the reduction of the impedance magnitude can be directly related to the changes in the physical properties of the functional mPEI layer. Since measurements were conducted with two CMUT chips connected in parallel, one of which had no modification of the surface and acted as the dummy chip for reference, any changes in temperature, fluctuations in pressure, flow rate variations and possible signal drift from the oscillator circuit can be discarded. When the sulfur and oxygen molecules are chemically absorbed through the bulk of the functional layer, it induces changes in its morphology leading to the increase in structural attenuation, while the interactions with CO_2_ molecules are on the surface of the functional layer and do not exhibit any morphological changes. Data from two different channels that include the shift in resonance frequency and magnitude of the impedance allowed to distinguish SO_2_ from CO_2_ by the difference in the impedance magnitude signal.

## 5. Conclusions

In this work, a Capacitive Micromachined Ultrasound Transducer based gas sensing system has been shown to be used for measurement of CO_2_ and SO_2_ concentrations in a dry nitrogen environment. The main resonance frequency and magnitude of the impedance spectra measurements were integrated together with DRIFTS spectroscopy system for real-time FT-IR measurements and an ex-situ Raman spectroscopy was used to get a better insight into the molecular interactions between gas molecules and used functional layer of methylated poly(ethylene)imine. It was found that the experimental setup for CO_2_ is reversible and the necessary modification layer can be used to determine the concentration of CO_2_, but due to the weak molecular interactions with mPEI it was only viable for lower concentration values, while the strong molecular bonds of SO_2_ to the mPEI layer gave a nearly linear response in all of the tested concentrations. However, the highly reactive nature of SO_2_ irreversibly changes the mPEI polymer physical structure creating a permanent shift in the resonance frequency measurement.

Importantly, during the initial tests of gas mixtures including both the CO_2_ and SO_2_ it was found that the changes of electroacoustic impedance magnitude can be detected when different gas interacts with the modification layer allowing the detection of the gas mixture. Constantly monitoring two individual parameters, it is possible to determine the characteristics of a gas mixture of CO_2_ and SO_2_ just from a CMUT chip modified with a single mPEI layer. Moreover, this fabricated CMUT gas sensing chip is not limited to using only one specific material, it was already shown from other researchers that the sensing area can be covered with various different materials that interact with other gases and optimizing these materials can potentially provide similar or better results [39,81] together with the optimization of manufacturing the CMUT chips. It has been shown by other researchers that the processing cost of CMUT could go down immensely using a single LOCOS process which does not require the expensive CMP step [28], reducing the production cost and achieving even better uniformity of the CMUT plate thickness. The acquired experimental data provides information regarding the next generation of CMUT devices functionalized with sensitive materials that can be used as multiple gas sensors.

## Figures and Tables

**Table 1:** Parameters used for materials.

Parameter	Value
Isolation layer, Hi SiO_2_	0.3 µm
SiO_2_ dielectric permittivity	3.7
Membrane thickness, Si	2 to 3 µm
Membrane elastic modulus, E	148 GPa
Membrane density, ρ	2329 kg/m^3^
Poisson’s ration	0.17729
Electrode, Ti/Au	0.2 µm (25 nm/175 nm)

**Table 2:** Parameters of manufactured CMUT device chips [59].

Parameters	Value
Membrane lateral dimensions, a	42 × 42 µm
Plate thickness, t	2 µm
Electrode thickness, t_e_	200 nm
Vacuum gap, g	150 nm
Number of cells (sensing elements)	1600
Resonance frequency, f_0_	16 MHz
Pull-in voltage, V_pin_	120 V

## Abbreviations

PEI	poly(ethylene-imine);
CMUT	capacitive micromachined ultrasonic transducer;
mPEI	methylated poly(ethylene-imine);
DRIFTS	diffuse reflectance infrared Fourier transform spectroscopy;
DMMP	dimethyl methyl phosphonate;
UV	ultraviolet;
SOI	silicon-on-insulator;
PECVD	plasma enhanced chemical vapor deposition;
THF	tetrahydrofuran;
MeOH	methanol;
Si_x_N_y_	silicon nitride (deposited using plasma enhanced chemical vapor deposition);
LOCOS	local oxidation of silicon;
SAW	surface acoustic wave;
FBAR	film bulk acoustic resonator;
QCM	quartz crystal microbalance;
DMMP	dimethyl methyl phosphonate;
PCB	printed circuit board;
USB	universal serial bus;
LOD	level of detection;
LOQ	level of quantification;
SNR	signal to noise ratio;
AC	alternating current;
DC	direct current;
RMSE	root mean square error;
FSI	fluid-solid interface.

## References

[1] Herrero M., Henderson B., Havlík P., Thornton P.K., Conant R.T., Smith P., Wirsenius S., Hristov A.N., Gerber P., Gill M. (2016). Greenhouse gas mitigation potentials in the livestock sector. Nat. Clim. Chang..

[2] Stavi I., Lal R. (2013). Agriculture and greenhouse gases, a common tragedy. A review. Agron. Sustain. Dev..

[3] Hynes S., Morrissey K., O’Donoghue C. (2013). Modelling Greenhouse Gas Emissions from Agriculture. Spatial Microsimulation for Rural Policy Analysis.

[4] Stevens B. (2017). Climate science: Clouds unfazed by haze. Nature.

[5] Stuart B. (2015). Infrared Spectroscopy. Kirk-Othmer Encyclopedia of Chemical Technology.

[6] Griffiths P. (1983). Fourier transform infrared spectrometry. Science.

[7] Subramanian A., Rodriguez-Saona L. (2009). Fourier Transform Infrared (FTIR) Spectroscopy. Infrared Spectroscopy for Food Quality Analysis and Contro.

[8] Cramers C.A., McNair H.M. (1983). Chapter 6 Gas chromatography. J. Chromatogr. Libr..

[9] Kopka J. (2006). Gas chromatography mass spectrometry. Biotechnology in Agriculture and Forestry.

[10] Jürgen U.K., Staudt R. (1976). Gravimetry in: Gas Adsorpt.

[11] Fanget S., Hentz S., Puget P., Arcamone J., Matheron M., Colinet E., Andreucci P., Duraffourg L., Myers E., Roukes M.L. (2011). Gas sensors based on gravimetric detection—A review. Sens. Actuators B Chem..

[12] Reddy P.R., Mohan B.C. (2012). Design and Analysis of Film Bulk Acoustic Resonator (FBAR) Filter for RF Applications. Int. J. Eng. Bus. Manag..

[13] Su Q.X., Kirby P., Komuro E., Imura M., Zhang Q., Whatmore R. (2001). Thin-film bulk acoustic resonators and filters using ZnO and lead-zirconium-titanate thin films. IEEE Trans. Microw. Theory Tech..

[14] Zhang Y., Luo J., Flewitt A.J., Cai Z., Zhao X. (2018). Film bulk acoustic resonators (FBARs) as biosensors: A review. Biosens Bioelectron.

[15] Doroodmand M.M., Sepehri S., Poorshamsi T. (2015). Room Temperature Quartz Crystal Microbalance-Based CO Sensor Using Commercial Piezoelectric Crystal Modified with Carbon Nanostructures. Sci. Adv. Mater..

[16] Qiao X., Zhang X., Tian Y., Meng Y. (2016). Progresses on the theory and application of quartz crystal microbalance. Appl. Phys. Rev..

[17] Sun P., Jiang Y., Xie G., Du X., Hu J. (2009). A room temperature supramolecular-based quartz crystal microbalance (QCM) methane gas sensor. Sens. Actuators B Chem..

[18] Casero E., Vázquez L., Parra-Alfambra A.M., Lorenzo E. (2010). AFM, SECM and QCM as useful analytical tools in the characterization of enzyme-based bioanalytical platforms. Analyst.

[19] Wang W., Hu H., Liu X., He S., Pan Y., Zhang C., Dong C. (2016). Development of a Room Temperature SAW Methane Gas Sensor Incorporating a Supramolecular Cryptophane A Coating. Sensors.

[20] Hashimoto K. (2012). Surface acoustic wave (SAW) devices. Ultrasonic Transducers.

[21] Wen W., Shitang H., Shunzhou L., Minghua L., Yong P. (2007). Enhanced sensitivity of SAW gas sensor coated molecularly imprinted polymer incorporating high frequency stability oscillator. Sens. Actuators B Chem..

[22] Mohanan A.A., Islam M.S., Ali S.H., Parthiban R., Ramakrishnan N. (2013). Investigation into mass loading sensitivity of sezawa wave mode-based surface acoustic wave sensors. Sensors.

[23] Subhashini S., Juliet A.V. (2013). CO_2_ Gas Sensor Using Resonant Frequency Changes in Micro-Cantilever. Computer Networks & Communications (NetCom).

[24] Dorsey K.L., Bedair S.S., Fedder G.K. (2014). Gas chemical sensitivity of a CMOS MEMS cantilever functionalized via evaporation driven assembly. J. Micromech. Microeng..

[25] Hsieh S., Hsieh S.L., Hsieh C., Lin P.C., Wu C.H. (2013). Label-Free Glucose Detection Using Cantilever Sensor Technology Based on Gravimetric Detection Principles. J. Anal. Methods Chem..

[26] Venstra W.J., Capener M.J., Elliott S.R. (2014). Nanomechanical gas sensing with nonlinear resonant cantilevers. Nanotechnology.

[27] Westwood J.N., Sauer V.T.K., Kwan J.K., Hiebert W.K., Sit J.C. (2014). Fabrication of nanoelectromechanical systems via the integration of high surface area glancing angle deposition thin films. J. Micromech. Microeng..

[28] Mølgaard M.J.G., Laustsen M., Jakobsen M.H., Andresen T.L., Thomsen E.V. (2018). Combined colorimetric and gravimetric CMUT sensor for detection of benzyl methyl ketone. Sens. Actuators B Chem..

[29] Lee H.J., Park K.K., Kupnik M., Oralkan O., Khuri-Yakub B.T. (2010). Highly sensitive detection of DMMP using a CMUT-based chemical sensor. Proceedings of the 2010 IEEE Sensors.

[30] Park K.K., Lee H., Kupnik M., Oralkan Ö., J-Ramseyer P., Lang H.P., Hegner M., Gerber C., Khuri-Yakub B.T. (2011). Capacitive micromachined ultrasonic transducer (CMUT) as a chemical sensor for DMMP detection. Sens. Actuators B Chem..

[31] Lee H.J., Park K.K., Oralkan O., Kupnik M., Khuri-Yakub B.T. (2008). CMUT as a chemical sensor for DMMP detection. Proceedings of the 2008 IEEE International Frequency Control Symposium.

[32] Virzonis D., Vanagas G., Ramanaviciene A., Makaraviciute A., Barauskas D., Ramanavicius A., Wen W., Kodzius R. (2014). Resonant gravimetric immunosensing based on capacitive micromachined ultrasound transducers. Microchim. Acta.

[33] Stedman Q., Park K.K., Khuri-Yakub B.T. (2014). Distinguishing chemicals using CMUT chemical sensor array and artificial neural networks. Proceedings of the 2014 IEEE International Ultrasonics Symposium.

[34] Park K.K., Lee H., Kupnik M., Khuri-Yakub B.T. (2011). Fabrication of Capacitive Micromachined Ultrasonic Transducers via Local Oxidation and Direct Wafer Bonding. J. Microelectromechanical Syst..

[35] Lee H.J., Park K.K., Kupnik M., Khuri-Yakub B.T. (2012). Functionalization layers for CO_2_ sensing using capacitive micromachined ultrasonic transducers. Sens. Actuators B Chem..

[36] Vanagas G., Barauskas D., Virzonis D. (2012). Study of the CMUT operation in microfluidic application. Proceedings of the 2012 IEEE International Ultrasonics Symposium.

[37] Barauskas D., Pelenis D., Sergalis G., Vanagas G., Mikolajunas M., Virzonis D., Baltrusaitis J. (2015). CMUT for high sensitivity greenhouse gas sensing. Proceedings of the 2015 IEEE International Ultrasonics Symposium (IUS).

[38] Barauskas D., Pelenis D., Virzonis D., Baltrus J.P., Baltrusaitis J. (2016). Greenhouse Gas Molecule CO_2_ Detection Using a Capacitive Micromachined Ultrasound Transducer. Anal. Chem..

[39] Stedman Q., Park K.K., Khuri-Yakub B.T. (2017). An 8-channel CMUT chemical sensor array on a single chip. Proceedings of the 2017 IEEE International Ultrasonics Symposium (IUS).

[40] Extrand C.W. (1994). Spin coating of very thin polymer films. Polym. Eng. Sci..

[41] Lawrence C.J. (1988). The mechanics of spin coating of polymer films. Phys. Fluids.

[42] Aziz F., Ismail A.F. (2015). Spray coating methods for polymer solar cells fabrication: A review. Mater. Sci. Semicond. Process..

[43] Hu Z., Zhang J., Xiong S., Zhao Y. (2012). Performance of polymer solar cells fabricated by dip coating process. Sol. Energy Mater. Sol. Cells..

[44] Tekin E., de Gans B.J., Schubert U.S. (2004). Ink-jet printing of polymers—from single dots to thin film libraries. J. Mater. Chem..

[45] Richardson J.J., Bjornmalm M., Caruso F. (2015). Technology-driven layer-by-layer assembly of nanofilms. Science.

[46] Bochenkov V.E., Sergeev G.B. (2010). Sensitivity, Selectivity and Stability of Gas-Sensitive Metal-Oxide Nanostructures. Metal Oxide Nanostructures and their Applications.

[47] Seok C., Mahmud M.M., Adelegan O., Zhang X., Oralkan O. (2016). A battery-operated wireless multichannel gas sensor system based on a capacitive micromachined ultrasonic transducer (CMUT) array. Proceedings of the 2016 IEEE SENSORS.

[48] Seok C., Mahmud M.M., Kumar M., Adelegan O.J., Yamaner F.Y., Oralkan O. (2018). A Low-Power Wireless Multichannel Gas Sensing System Based on a Capacitive Micromachined Ultrasonic Transducer (CMUT) Array. IEEE Internet Things J..

[49] Yoon I., Eom G., Lee S., Kim B.K., Kim S.K., Lee H.J. (2019). A Capacitive Micromachined Ultrasonic Transducer-Based Resonant Sensor Array for Portable Volatile Organic Compound Detection with Wireless Systems. Sensors.

[50] Wygant I.O., Kupnik M., Khuri-Yakub B.T. (2008). Analytically calculating membrane displacement and the equivalent circuit model of a circular CMUT cell. Proceedings of the 2008 IEEE Ultrasonics Symposium.

[51] Oguz H.K., Atalar A., Koymen H. (2013). Equivalent circuit-based analysis of CMUT cell dynamics in arrays. IEEE Trans. Ultrason. Ferroelectr. Freq. Control.

[52] Lohfink A., Eccardt P.C. (2005). Linear and nonlinear equivalent circuit modeling of CMUTs. IEEE Trans. Ultrason. Ferroelectr. Freq. Control.

[53] Oguz H.K., Olcum S., Senlik M.N., Atalar A., Koymen H. (2009). A novel equivalent circuit model for CMUTs. Proceedings of the 2009 IEEE International Ultrasonics Symposium.

[54] Aydogdu E., Ozgurluk A., Oguz H.K., Atalar A., Kocabas C., Koymen H. (2012). Nonlinear equivalent circuit model for circular CMUTs in uncollapsed and collapsed mode. Proceedings of the 2012 IEEE International Ultrasonics Symposium.

[55] Jin X.C., Ladabaum I., Degertekin F.L., Calmes S., Khuri-Yakub B.T. (1999). Fabrication and characterization of surface micromachined capacitive ultrasonic immersion transducers. J. Microelectromechanical Syst..

[56] Ladabaum I., Jin X., Soh H.T., Atalar A., Khuri-Yakub B.T. (1998). Surface micromachined capacitive ultrasonic transducers. IEEE Trans. Ultrason. Ferroelectr. Freq. Control.

[57] Molgaard M.J.G., Laustsen M., Thygesen I.L., Jakobsen M.H., Andresen T.L., Thomsen E.V. (2017). Combined colorimetric and gravimetric CMUT sensor for detection of phenylacetone. Proceedings of the 2017 IEEE International Ultrasonics Symposium (IUS).

[58] Molgaard M.J.G., Hansen J.M.F., Jakobsen M.H., Thomsen E.V. (2018). Sensitivity Optimization of Wafer Bonded Gravimetric CMUT Sensors. J. Microelectromechanical Syst..

[59] Barauskas D., Park S.J., Pelenis D., Vanagas G., Lee J.J., Virzonis D., Jones C.W., Baltrusaitis J. (2019). CO_2_ and SO_2_ interactions with methylated poly(ethyleneimine) functionalized Capacitive Micromachined Ultrasonic Transducers (CMUTs): Gas sensing and degradation mechanism. ACS Appl. Electron. Mater..

[60] Yongli H., Ergun A.S., Haggstrom E., Badi M.H., Khuri-Yakub B.T. (2003). Fabricating capacitive micromachined ultrasonic transducers with wafer-bonding technology. J. Microelectromechanical Syst..

[61] Park K.K., Lee H.J., Kupnik M., Oralkan Ö., BTKhuri-Yakub Oralkan O., Khuri-Yakub B.T. (2008). Fabricating capacitive micromachined ultrasonic transducers with direct wafer-bonding and LOCOS technology. Proceedings of the 2008 IEEE 21st International Conference on Micro Electro Mechanical Systems.

[62] Williams K.R., Gupta K., Wasilik M. (2003). Etch rates for micromachining processing-part II. J. Microelectromechanical Syst..

[63] Kang J.K., Musgrave C.B. (2002). The mechanism of HF/H_2_O chemical etching of SiO_2_. J. Chem. Phys..

[64] Vanheusden K., Stesmans A. (1991). Chemical etch rates in HF solutions as a function of thickness of thermal SiO_2_ and buried SiO_2_ formed by oxygen implantation. J. Appl. Phys..

[65] Somashekhar A. (1996). Etching SiO_2_ Films in Aqueous 0.49% HF. J. Electrochem. Soc..

[66] Christiansen T.L., Hansen O., Johnsen M.D., Lohse J.N., Jensen J.A., Thomsen E.V. (2013). Void-free direct bonding of CMUT arrays with single crystalline plates and pull-in insulation. Proceedings of the 2013 IEEE International Ultrasonics Symposium (IUS).

[67] Henry M.D., Welch C., Scherer A. (2009). Techniques of cryogenic reactive ion etching in silicon for fabrication of sensors. J. Vac. Sci. Technol. A Vac. Surf. Film..

[68] Pruessner M.W., Rabinovich W.S., Stievater T.H., Park D., Baldwin J.W. (2007). Cryogenic etch process development for profile control of high aspect-ratio submicron silicon trenches. J. Vac. Sci. Technol. B Microelectron. Nanom. Struct..

[69] Zhu G., Carrillo M.Y.J., Sujan A., Okonkwo C.N., Park S., Sumpter B.G., Jones C.W., Lively R.P. (2018). Molecular blends of methylated-poly(ethylenimine) and amorphous porous organic cages for SO_2_ adsorption. J. Mater. Chem. A.

[70] Tailor R., Abboud M., Sayari A. (2014). Supported Polytertiary Amines: Highly Efficient and Selective SO_2_ Adsorbents. Environ. Sci. Technol..

[71] Al-Marri M.J., Al-Saad K.A., Saad M.A., Cortes D.J., Khader M.M. (2017). Thermodynamics of CO_2_ Adsorption on Polyethyleneimine Mesoporous Silica and Activated Carbon. J. Phys. Chem. Biophys..

[72] Barauskas D., Pelenis D., Viržonis D., Sapeliauskas E. Capacitive micromachined ultrasound transducer for greenhouse gas detection. Proceedings of the 11th International Conference Intelligent Technologies in Logistics and Mechatronics Systems.

[73] Khader M.M., Al-Marri M.J., Ali S., Qi G., Giannelis E.P. (2015). Adsorption of CO_2_ on Polyethyleneimine 10k—Mesoporous silica Sorbent: XPS and TGA Studies. Am. J. Anal. Chem..

[74] Andreoli E., Cullum L., Barron A.R. (2015). Carbon Dioxide Absorption by Polyethylenimine-Functionalized Nanocarbons: A Kinetic Study. Ind. Eng. Chem. Res..

[75] Goeppert A., Czaun M., May R.B., Prakash G.K.S., Olah G.A., Narayanan S.R. (2011). Carbon dioxide capture from the air using a polyamine based regenerable solid adsorbent. J. Am. Chem. Soc..

[76] Lee H.J., Park K.K., Oralkan Ö., Kupnik M., Khuri-Yakub B.T. (2014). A Multichannel Oscillator for a Resonant Chemical Sensor System. IEEE Trans. Ind. Electron..

[77] Mahmud M.M., Li J., Lunsford J.E., Zhang X., Yamaner F.Y., Nagle H.T., Oralkan O. (2014). A low-power gas sensor for environmental monitoring using a capacitive micromachined ultrasonic transducer. Proceedings of the Sensors, 2014 IEEE.

[78] Koymen H., Atalar A., Aydogdu E., Kocabas C., Oguz H., Olcum S., Ozgurluk A., Unlugedik A. (2012). An improved lumped element nonlinear circuit model for a circular CMUT cell. IEEE Trans. Ultrason. Ferroelectr. Freq. Control.

[79] Lee H.J., Park K.K., Cristman P., Oralkan O., Kupnik M., Khuri-Yakub B.T. (2009). A Low-Noise Oscillator based on a Multi-Membrane CMUT for High Sensitivity Resonant Chemical Sensors. Proceedings of the 2009 IEEE 22nd International Conference on Micro Electro Mechanical Systems.

[80] Briggs A.G. (1970). Vibrational frequencies of sulfur dioxide. Determination and application. J. Chem. Educ..

[81] Mahmud M.M., Kumar M., Zhang X., Yamaner F.Y., Nagle H.T., Oralkan O. A capacitive micromachined ultrasonic transducer (CMUT) array as a low-power multi-channel volatile organic compound (VOC) sensor. Proceedings of the 2015 IEEE SENSORS, IEEE.

